# Supplementary material for: Microbial Synthesis of Heme b: Biosynthetic Pathways, Current Strategies, Detection, and Future Prospects
Source: Molecules. 2023 Apr 21;28(8):3633. doi: 10.3390/molecules28083633 (PMC10144233; doi:10.3390/molecules28083633)
Supplement: Supplementary file 1 [file molecules-28-03633-s001.zip › molecules-2281374-supplementary.pdf]

Review

Microbial Synthesis of Heme *b*: Biosynthetic Pathways, Current Strategies, Detection, and Future Prospects

Qiuyu Yang [1,2], Juntao Zhao [1,2], Yangyang Zheng [1,2], Tao Chen [1,2] and Zhiwen Wang [1,2,*]

1 Frontier Science Center for Synthetic Biology and Key Laboratory of Systems Bioengineering (Ministry of Education), Tianjin University, Tianjin 300072, China
2 SynBio Research Platform, Collaborative Innovation Center of Chemical Science and Engineering (Tianjin), School of Chemical Engineering and Technology, Tianjin University, Tianjin 300072, China
* Correspondence: zww@tju.edu.cn; Tel.: +86-22-85356605

Supplementary Information

Table S1. Calculated $\Delta G^{0'}$ values for the production of heme *b*.

Enzymes	Reactions (Substrates → Products)	$\Delta G^{0'}$ (kJ/mol)
AlaS	Succinyl-CoA + Gly + ATP → 5-ALA + CoA + CO_2 + AMP + PPi	25.73
GluTS	L-Glu + Glu^{tRNA} + ATP →L-Glu-Glu^{tRNA} + AMP + PP_i	-369.36
GluTR	L-Glu-Glu^{tRNA} + NADPH + H^+ →GSA+ Glu^{tRNA} + $NADP^+$	372.94
GsaM	GSA → 5-ALA	-6.11
PbgS	2 5-ALA → PBG + 2 H_2O	-141.13
HmbS	4 PBG + H_2O → HMB + 4 NH_3	-207.52
UroS	HMB → UPG III + H_2O	-55.13
UroD	UPG III → CPG III + 4 CO_2	-14.12
CgdC	CPG III + 2 SAM → PPG IX + 2 5′-deoxyadenosine + 2 L-methionine + 2 CO_2	-115.27
PgoX	PPG IX + 3 O_2 →PP IX + 3 H_2O_2	-622.83
PpfC	PP IX + Fe^{2+} → Heme + 2 H^+	244.65
CgoX	CPG III + 3 O_2 →CP III + 3 H_2O_2	-662.83
CpfC	CP III + Fe^{2+} → coproporphyrin III + 2 H^+	244.65
ChdC	coproporphyrin III + 2 H_2O_2 → Heme + 2 CO_2 + 4 H_2O	-562.79
